# Evaluation of the ability of the Clinical Treatment Score at 5 years (CTS5) compared to other risk stratification methods to predict the response to an extended endocrine therapy in breast cancer patients

**DOI:** 10.1007/s12282-021-01258-5

**Published:** 2021-05-03

**Authors:** Andrea Villasco, Francesca Accomasso, Marta D’Alonzo, Francesca Agnelli, Piero Sismondi, Nicoletta Biglia

**Affiliations:** 1 Academic Division of Gynecology and Obstetrics, Mauriziano Umberto I Hospital, University of Turin, Via Magellano 1, 10128 Turin, Italy; 2 School of Medicine, University of Turin, Turin, Italy

**Keywords:** Estrogen receptor-positive, Breast cancer, Late distant recurrence, CTS5, Extended endocrine therapy

## Abstract

**Purpose:**

Extension of adjuvant endocrine therapy (ET) reduces the risk of recurrence in women diagnosed with ER-positive breast cancers, but a significant benefit is unlikely to happen to all individual patients. This study is aimed at evaluating the ability of different clinical late distant recurrence (LDR) risk stratification methods and in particular the clinical treatment score at 5 years (CTS5) to predict the response to extended adjuvant ET.

**Methods:**

783 patients diagnosed with ER+ BC between 1988 and 2014 at Umberto I Hospital of Turin, of which 180 received an extended adjuvant ET, were retrospectively selected. They were stratified according to pT, pN, disease stage, tumor grade, Ki67 level, progesterone receptor status and CTS5. The primary endpoint was LDR rate. LDR rates according to ET duration were confronted in each subgroup.

**Result:**

The median duration of extended ET was 7 years (6–10). Median follow-up from diagnosis was 9 years (6–26). Retrospective risk stratification according to tumor size, nodal status, disease stage, tumor grade, Ki67 level, and progesterone receptor status did not appear to be able to predict the response to extended ET. In the CTS5 high-risk subgroup instead, the risk of developing an LDR was significantly lower in the patients who underwent extended ET compared to standard ET (HR 0.37, 95% CI 0.15–0.91), while no significant benefit was demonstrated for low and intermediate-risk patients.

**Conclusions:**

Risk stratification according to CTS5 appeared to be predictive of the response to extended endocrine therapy in our population of real-life pre and postmenopausal patients.

## Introduction

Breast cancer is the most frequent neoplasia in women worldwide, and about 80% of new diagnoses are estrogen receptor-positive (ER+) tumors [1]. With current surgical and medical treatment, the results both in terms of disease recurrence and short-term survival for early ER+ breast cancer patients are excellent [2]. 5 years of adjuvant endocrine therapy (ET) reduce mortality (by 30% with five years of Tamoxifen and 40% with an aromatase inhibitor (AI) in postmenopausal women) [3] and hence represent the gold standard treatment for these patients [4]. Nevertheless, up to 50% of distant recurrences (DR) show up after the completion of adjuvant endocrine therapy. Numerous studies on extended ET (e-ET) have shown a significant improvement in disease outcomes, but their results are hardly applicable to the current clinical scenario. In postmenopausal women, extended therapy appears to be most beneficial in patients treated with Tamoxifen for 5 years who either continue Tamoxifen or switch to an AI-based regimen. Such schedules have now been long abandoned in favor of upfront AI therapies that have already shown superiority over Tamoxifen. On the contrary, the benefits of extending a 5-year course of adjuvant AI appear to be less substantial, except for specific subgroups of patients at higher risk of recurrence. For premenopausal women instead, data on extended therapy is limited and will not be applicable in the near future since the SOFT [4] and TEXT [5] trials have now revolutionized the clinical practice. On these premises, the selection of the patients at higher recurrence risk deemed to get the most benefit from an extended endocrine therapy has now become a prominent concern. Many molecular profiles have been tested and proved to provide prognostic information on LDR risk after five years of ET, even though they were created to be used at diagnosis [6]. Contrariwise, the Clinical Treatment Score at 5 years (CTS5), which integrates four clinicopathological variables, was specifically developed to estimate the LDR risk after five years of adjuvant endocrine therapy for ER+ breast cancer. It was created and validated on the ATAC and BIG 1–98 cohorts of postmenopausal patients [7] and its prognostic ability has now been externally confirmed on different cohorts of pre and postmenopausal patients [8–11]. Apart from the prognostic information, limited data is available on the ability of these tools to predict the response to the extended ET. In particular, only the Breast Cancer Index (BCI) was shown to be *predictive* of the response to extended ET [12, 13].

This study aimed at evaluating the ability of different risk stratification methods (tumor size, nodal involvement, stage of disease, tumor grade, Ki67 level, progesterone receptor status and CTS5) carried out retrospectively to predict the response to an extended ET in a sample of pre and postmenopausal real-life patients.

## Materials and methods

Between 1988 and 2014, 2806 patients were treated for breast cancer at Mauriziano Umberto I Hospital of Turin. They underwent surgery, chemotherapy, radiation therapy, and endocrine therapy according to the guidelines in force at the time.

From this data set, we selected only patients with invasive tumors, complete clinicopathological data, and full immunohistochemical characterization. Tumors were considered estrogen receptor-positive when the staining involved > 1% of the specimen cancer cells. Endocrine therapy was prescribed, according to the guidelines, to all patients with tumors fulfilling this criterion.

We retained for this analysis 783 ER-positive, HER2 negative patients after excluding 101 patients who experienced a disease recurrence during the first five years of ET and 26 patients who experienced a late locoregional recurrence without evidence of distant disease. One-hundred-eighty patients of the 783 selected underwent an e-ET. The patient selection process is outlined in Fig. 9.

The 783 remaining patients that were included in the analysis underwent a retrospective LDR stratification according to tumor size, nodal status, stage of disease, tumor grade, Ki67 level, progesterone receptor status and CTS5. CTS5 score was calculated applying the final validated formula:

$${\text{CTS5}} = 0.{438} \times {\text{nodes}} + 0.{988} \times \left( {0.0{93} \times {\text{size}} - 0.00{1} \times {\text{size}}^{{2}} + 0.{375} \times {\text{grade}} + 0.0{17} \times {\text{age}}} \right).$$

Patients were categorized in three risk groups based on the score obtained: low- (CTS5 < 3.13), intermediate- (3.13–3.86), and high-risk (> 3.86).

In each subgroup of the different risk stratifications, the patients were further divided according to endocrine therapy duration: standard 5 years course or extended regimen.

Extended endocrine therapy prescription was not the standard clinical practice in the period considered. The prescription was made according to the evaluation of the clinician of individual risk factors and in agreement with patients, with duration to be determined on the patients’ tolerance to treatment.

The primary endpoint of this study was the late distant recurrence (LDR) rate.

### Statistical analysis

Categorical variables were confronted using Chi-square test. Two-sided *p* values < 0.05 were considered statistically significant. Survival analysis was performed with the Kaplan Meier method; the differences between the curves were estimated with the log-rank test. Hazard ratios were obtained with the Cox regression model and reported at the 95% confidence interval.

## Results

Out of the 783 women enrolled for this analysis, 533 (68.1%) were postmenopausal and 250 (31.9%) premenopausal. Patients’ characteristics are shown in Table 1. Median follow-up after standard 5 years of adjuvant ET was 4 years (range 1–21). The 42.9% of our patients had at least a five-year follow up, 18.9% a 3–4-year follow-up, the 38.2% 2 or less years of follow-up. An Aromatase Inhibitors-based endocrine therapy was given upfront to 379 (71.1%) postmenopausal women, while Tamoxifen + Ovarian Function Suppression (OFS) was the preferred choice for premenopausal women (161/250, 64.4%) (Table 2). Extended endocrine therapy was prescribed to 180 patients, 107/533 (20.1%) postmenopausal and 73/250 (29.2%) premenopausal. For these patients, the median total duration of endocrine therapy was 7 years (6–10). Out of the 73 premenopausal patients who have prescribed an extended ET, 34 (46.6%) switched from Tamoxifen ± OFS to an AI, while the remaining 39 (53.4%) continued Tamoxifen without OFS (15 patients previously on Tamoxifen alone and 24 patients previously on OFS). Concerning postmenopausal patients, 83 (77.6%) continued on the same AI administered during the previous five years while 20 (18.7%) switched from Tamoxifen to an AI. Only four postmenopausal patients continued Tamoxifen alone in the extended setting.

**Table 1:** Patients' characteristics

	5 years of ET	Extended ET
Total	603 (77.0%)	180 (23.0%)
Menopausal Status		
Premenopausal	177 (70.8%)	73 (29.2%)
Postmenopausal	426 (79.9%)	107 (20.1%)
Median age		
Premenopausal	45 (26–54)	45 (27–54)
Postmenopausal	67 (51–91)	64 (51–83)
Median ET duration		
Premenopausal	5	7 (6–10)
Postmenopausal	5	7 (6–10)
Surgery		
BCS	425 (78.8%)	114 (21.2%)
Mastectomy	178 (73.0%)	66 (27.0%)
LDR rate		
Total events	54 (9%)	9 (5%)

**Table 2:**
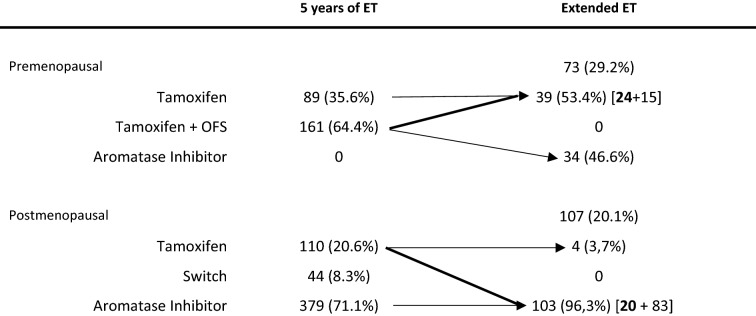
Endocrine therapy

*Arrows and numbers in bold: patients switching from one type of ET to the other, detail in text

Global LDR rate was 8%: 9% in the 5 years ET group and 5% in the extended ET group (*p* = 0.08) (Table 1). Median follow-up among patients who experienced an LDR was similar in both groups (5y ET: 10 years [6–26], extended ET: 10 years [6–16]).

To identify the patients at higher risk of LDR, a retrospective stratification was carried out according to CTS5 and other parameters commonly used in the clinical practice: tumor size (T1, T2, T3 or higher), nodal involvement (pN0, pN1, pN2-3), stage of the disease (Stages I, II, III), tumor grade (low, intermediate, high), Ki67 (low [< 20%], high [> 20%]), and progesterone receptor status (negative [< 1%], positive [> 1%]). The prescription of extended endocrine therapy was significantly associated to worse clinical and pathological features. We observed a higher prescription rate in patients with larger tumors (38.5% of pT3–4 vs 26.9% of pT2 vs 19.5% of pT1, *p* = 0.01), patients with greater nodal involvement (34.3% of pN2–3 vs 30.6% of pN1 vs 16.9% of pN0, *p* < 0.001), patients with a higher clinical stage (35.3% of Stage III vs 25.9% of Stage II vs 15.5% of Stage I, *p* < 0.001), patients with higher Ki67 (33.2% of Ki67 > 20% vs 16.5% of Ki67 < 20%), and patients with higher histologic grade (32.6% of G3 vs 17.8% of G2 vs 10.3% of G1, *p* < 0.001). Instead, PgR status did not appear to affect e-ET prescription rate (18.7% of PgR-negative vs 23.4% PgR-positive patients, *p* = 0.39). After obtaining the CTS5 score for all patients, we observed that an extended ET was also prescribed in a significantly higher proportion to patients categorized as High-Risk (31.9% vs 14.9% low-risk, *p* < 0.001). Details are shown in Table 3.

**Table 3:** Extended ET according to pT, pN, Stage, ki67, PgR status and histologic tumor grade

	5 years of ET	Extended ET	*p* value
Tumor size			
pT1	367 (80.5%)	89 (19.5%)	
pT2	220 (73.1%)	81 (26.9%)	0.01
pT3-4	16 (61.5%)	10 (38.5%)	
Positive nodes			
pN0	384 (83.1%)	78 (16.9%)	< 0.001
pN1	154 (69.4%)	68 (30.6%)	
pN2–3	65 (65.7%)	34 (34.3%)	
Stage			< 0.001
Stage I	277 (84.5%)	51 (15.5%)	
Stage II	249 (74.1%)	87 (25.9%)	
Stage III	77 (64.7%)	42 (35.3%)	
Ki67			
Low (< 20%)	400 (83.5%)	79 (16.5%)	< 0.001
High (> 20%)	203 (66.8%)	101 (33.2%)	
Histologic Grade			
G1	113 (89.7%)	13 (10.3%)	
G2	263 (82.2%)	57 (17.8%)	< 0.001
G3	227 (67.4%)	110 (32.6%)	
PgR status			
Negative (< 1%)	61 (81.3%)	14 (18.7%)	0.390
Positive (> 1%)	542 (76.6%)	166 (23.4%)	
CTS5			
Low risk	240 (85.1%)	42 (14.9%)	
Intermediate risk	179 (77.5%)	52 (22.5%)	< 0.001
High risk	184 (68.1%)	86 (31.9%)	

**Fig. 1:**
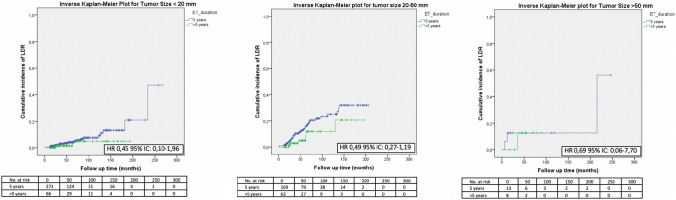
LDR risk according to ET duration and tumor size

**Fig. 2:**
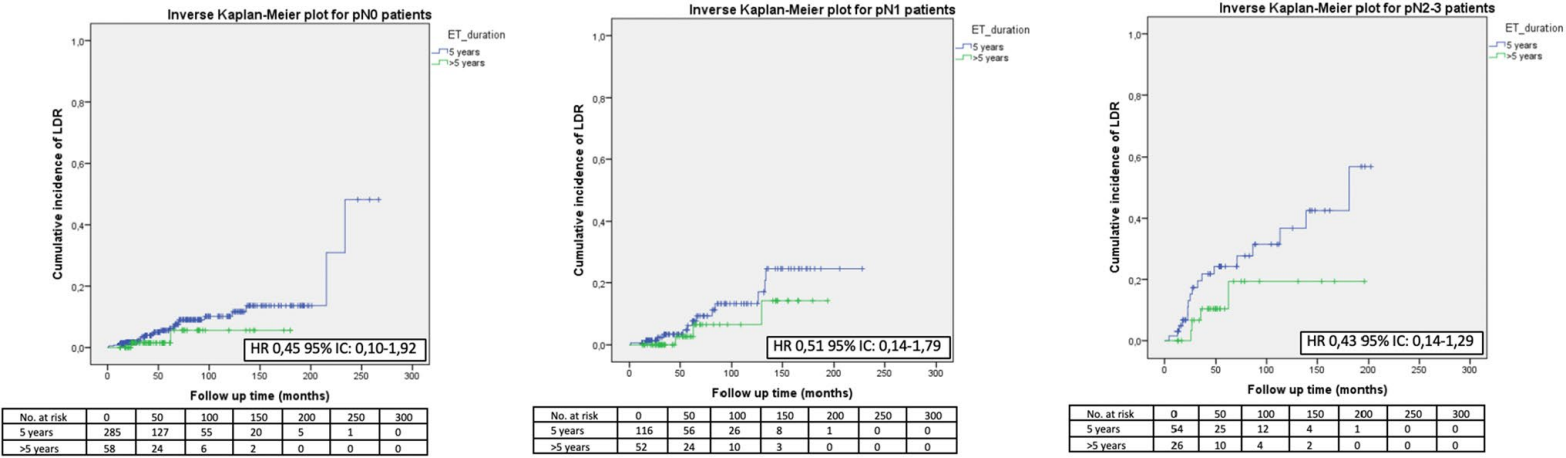
LDR risk according to ET duration and nodal status

**Fig. 3:**
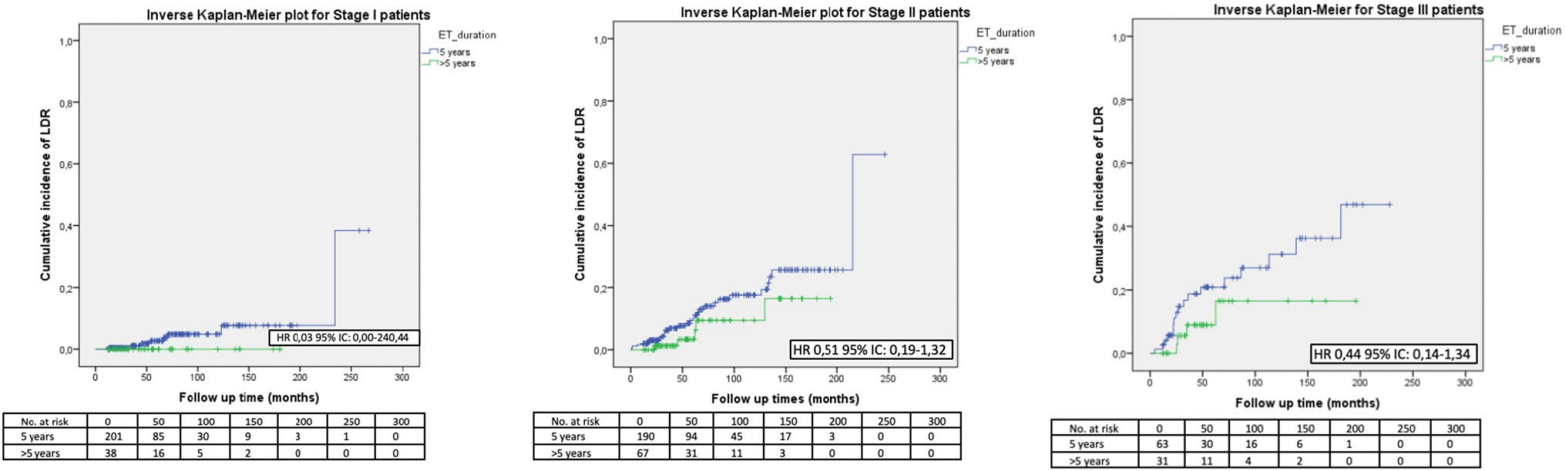
LDR risk according to ET duration and stage of disease

**Fig. 4:**
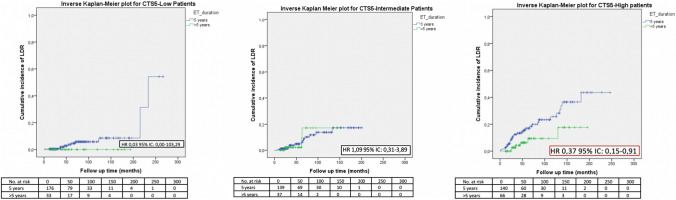
LDR risk according to ET duration and CTS5

**Fig. 5:**
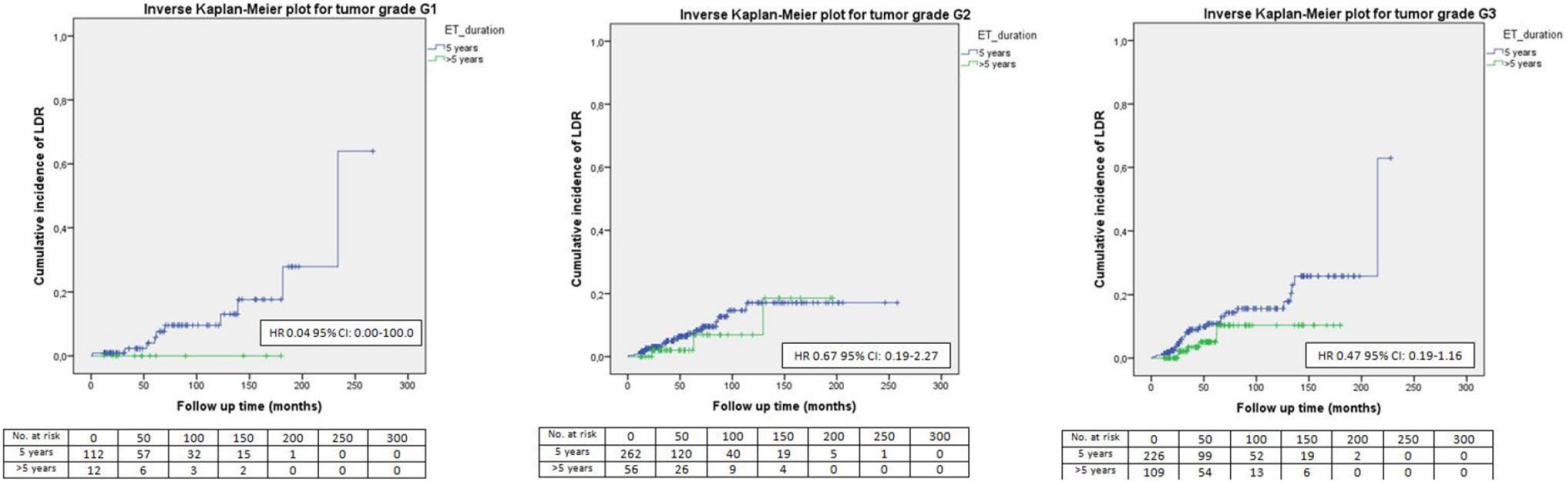
LDR risk according to ET duration and tumor grade

**Fig. 6:**
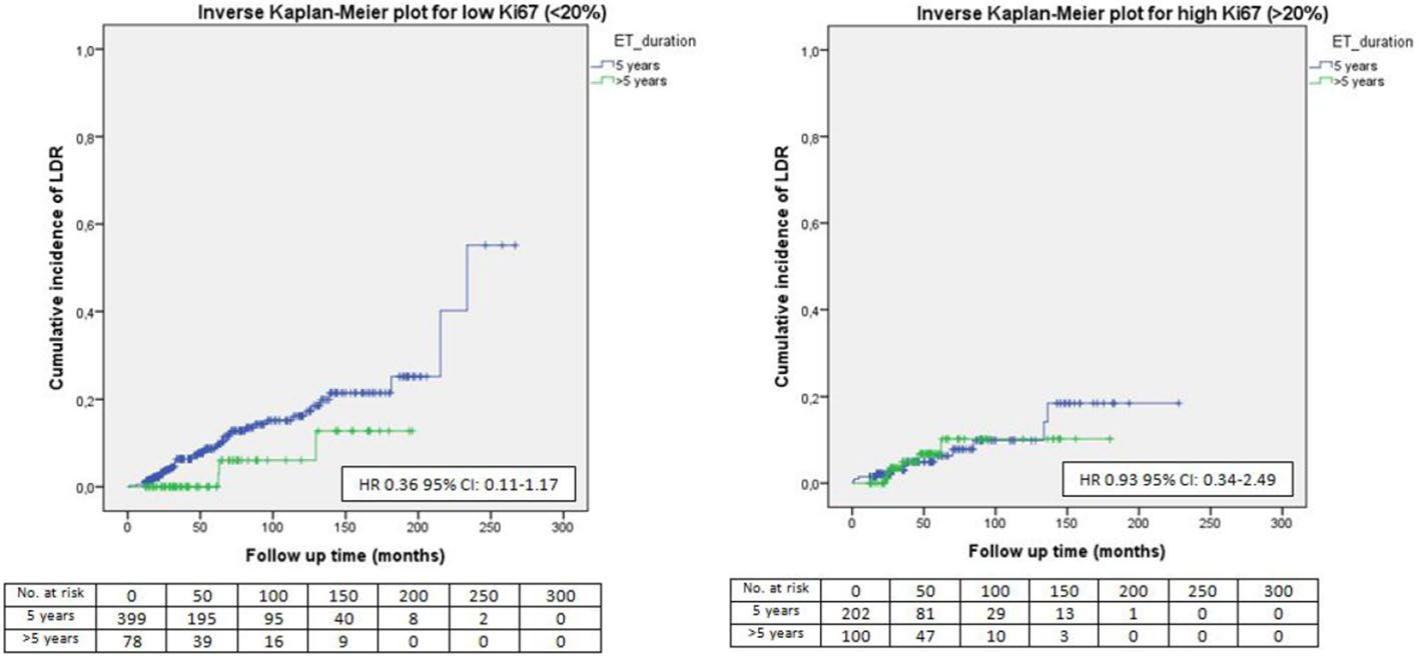
LDR risk according to ET duration and Ki67

**Fig. 7:**
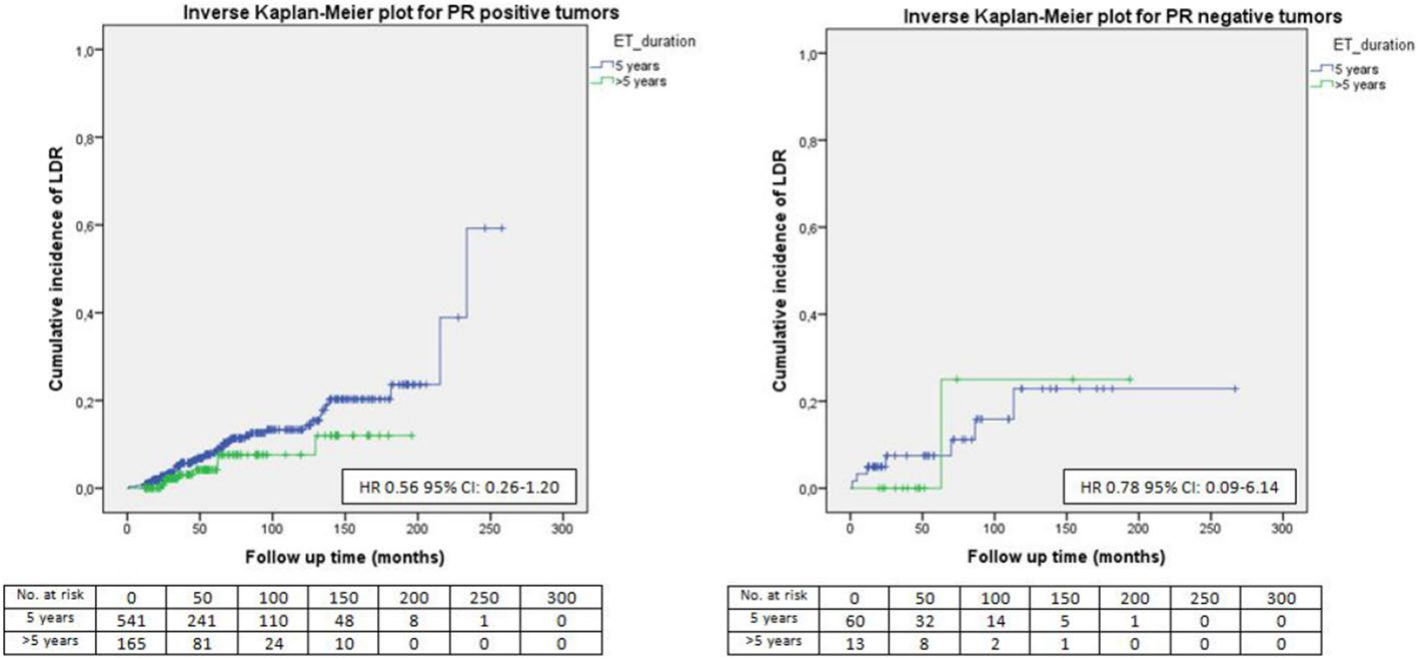
LDR risk according to ET duration and PgR status

No significant difference in terms of LDR risk could be observed between patients undergoing standard versus extended ET when they were categorized according to tumor size (Fig. 1), nodal involvement (Fig. 2), stage of disease (Figs. 3, 4), tumor grade (Fig. 5), Ki67 level (Fig. 6) and progesterone receptor status (Fig. 7).

On the contrary, risk stratification according to CTS5 was shown to be predictive of the response to the extended endocrine therapy (Fig. 4). In particular, the risk of developing a late distant recurrence with extended endocrine therapy was significantly lower in patients categorized as high-risk (HR 0.37; 95% CI 0.15–0.91, *p* = 0.04) while no significant difference was outlined for low-risk patients (HR 0.03; 95% CI 0.00–103.29) and intermediate-risk patients (HR 1.09; 95% CI 0.31–3.89).

**Fig. 8:**
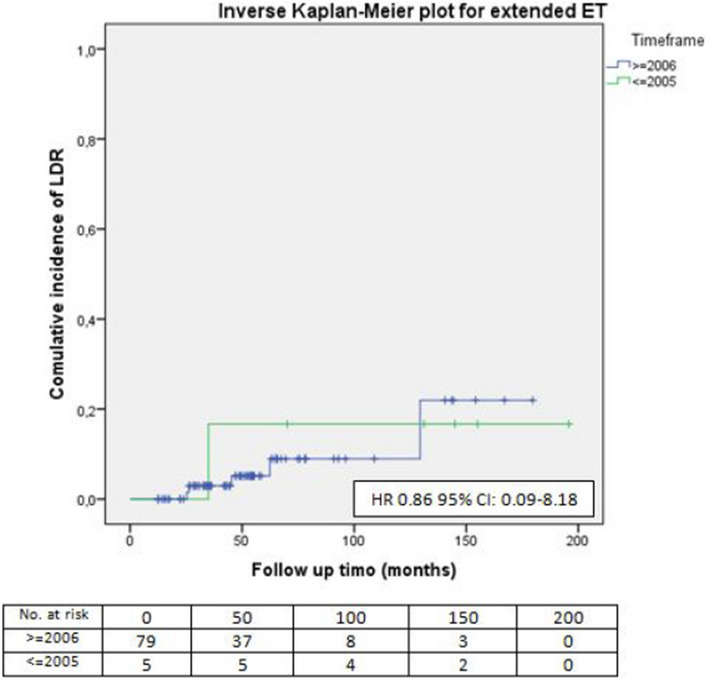
LDR risk for CTS5 high-risk patients who underwent extended ET according to time of surgery

**Fig. 9:**
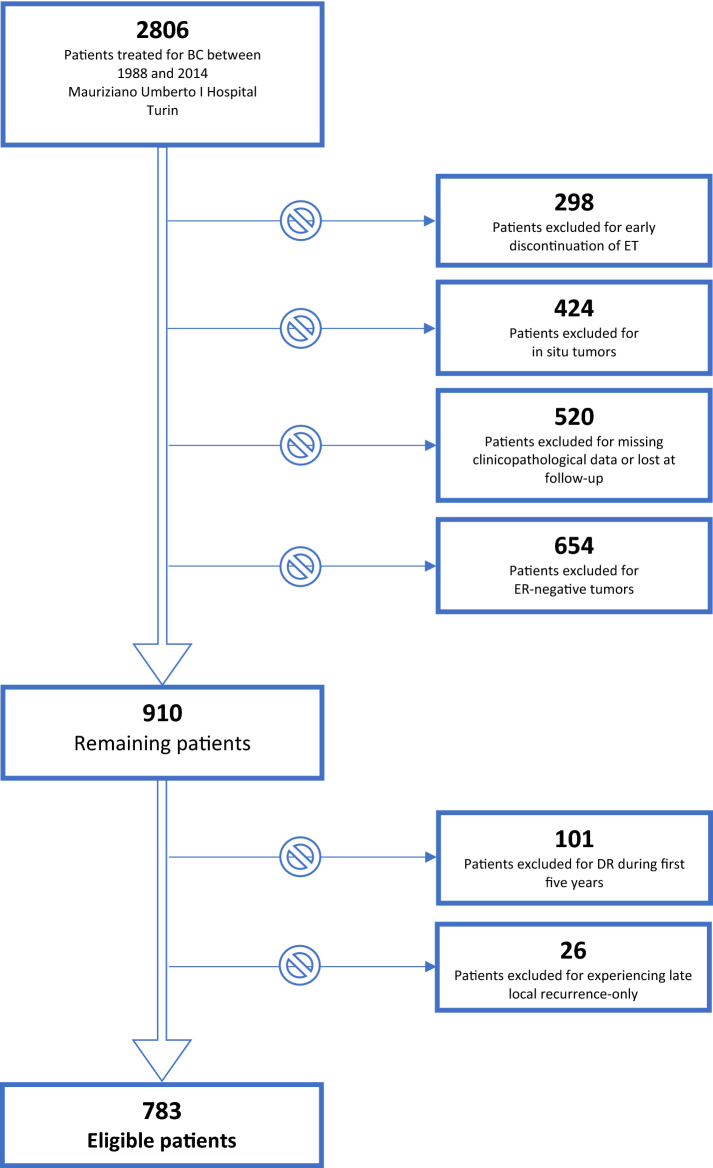
Patients selection process

Among patients categorized as high risk by CTS5, adjuvant chemotherapy and radiotherapy were comparable both in the extended and in the standard ET groups. The majority of the patients prescribed an extended endocrine regimen underwent surgery after 2005 (Table 4), but no differences in terms of LDR risk could be outlined when compared to patients who underwent an extended ET after surgery performed before that year (HR 0.86, 95% CI 0.09–8.18) (Figs. 8, 9).

**Table 4:** CTS5 high risk patients

	5 years of ET	Extended ET	*p* value
Chemotherapy			
Yes	141 (65.9%)	73 (34.1%)	0.14
No	43 (76.8%)	13 (23.2%)	
Radiotherapy			
Yes	134 (66.7%)	67 (33.3%)	0.45
No	50 (72.5%)	19 (27.5%)	
Timeframe			
≤ 2005	87 (84.5%)	16 (15.5%)	< 0.001
≥ 2006	97 (58.1%)	70 (41.9%)

## Discussion

A metanalysis published by Pan H. et al. including more than 60,000 women has shown that the risk of distant recurrence persists for at least 20 years from the diagnosis of an ER+ breast cancer [14]. There is now evidence that this risk can be reduced by extending the course of endocrine therapy, but a significant benefit is unlikely to happen to all individual patients.

Many studies have been conducted on this topic: eight RCTs involving more than 17,000 patients were recently included in a metanalysis by Corona et al., which confirmed the beneficial effect of the extended endocrine therapy on DFS but not on OS [15]. The protection offered by the extended endocrine therapy comes along with an increased incidence of therapy-related side effects: endometrial cancer and thromboembolic disease for Tamoxifen, cardiovascular events, and bone fractures for AIs [16]. These results raise several questions when it comes to recommending extended adjuvant endocrine therapy, such as those concerning patient selection, the agent of choice, and the duration of treatment.

Several multigene assays have been proven to provide prognostic information on the risk of LDR. A recently published pan-genomic analysis by the TransATAC study group compared the performance of six prognostic signatures: CTS, IHC4, BCI prognostic score, Oncotype Dx, Prosigna, and EndoPredict. While all tools were able to provide prognostic information on the risk of early recurrence, only the BCI score, Prosigna, and EndoPredict demonstrated a significant ability to stratify patients for LDR risk, above and beyond clinical parameters [17]. The prognostic performance was weaker among patients with nodal involvement, once more underlying the importance of this clinical variable also in the long term.

So far, little is known about the magnitude of the benefit associated with the extended endocrine treatment according to the risk stratification carried out with the prognostic tools.

Historically, the decision to propose an extended endocrine regimen was based on the only two clinical variables shown to be independently associated with the risk of late recurrence: tumor size and lymph node burden [18]. More recently, an algorithm integrating four clinicopathological variables, the Clinical Treatment Score at 5 years (CTS5), was shown to be prognostic for late distant recurrence, thus providing useful information to help to tailor the prescription of extended ET. This study was aimed at investigating whether the CTS5-based risk stratification could be helpful in choosing patients more likely to benefit from extended endocrine therapy. The analysis was conducted on a retrospective cohort of 783 patients, of which 180 were prescribed extended endocrine therapy. We found that neither tumor size nor nodal involvement, stage of the disease, tumor grade, Ki67 level, and progesterone receptor status could individually predict the response to such treatment. On the contrary, the risk stratification according to CTS5-based showed a significant benefit of the extended endocrine therapy in high-risk patients with a 60% reduction in the risk of developing an LDR. This suggests that the CTS5 can help in selecting the patients more likely to profit from the treatment. No benefit from extended ET administration has been observed in low and intermediate-risk patients. It is to be noted that this result was obtained with a median duration of extended endocrine therapy of only 7 years, while the standard consensus considers 10 years of endocrine therapy the ideal objective [19]. It has recently been observed that recent advantages in diagnostics and treatment could have concurred to the better outcomes of the patients treated with extended endocrine therapy [20]; however, we did not observe different LDR risks confronting patients who underwent an extended endocrine therapy after surgery performed before versus after 2005. To our knowledge, this is the first study to evaluate the predictive ability of the CTS5. Only two other prospective-retrospective studies, both involving a component of Breast Cancer Index prognostic score (BCI H/I), have conducted a similar analysis. BCI (H/I) predictive ability was initially demonstrated in the NCIC-CTG MA.17 RCT cohort (*N* = 249, 60% N +). Patients categorized as BCI (H/I)-High had a significantly improved outcome with extended letrozole treatment versus placebo: a 67% reduction in risk of recurrence (OR 0.35; 95% CI 0.16–0.75; *p* = 0.007), while patients with BCI (H/I)-low did not have a statistically significant decrease in late recurrence when treated with extended endocrine therapy (OR 0.68; 95% CI 0.31–1.52; *p* = 0.35). More recently, Bartlett and Sgroi tested the BCI prognostic index on 583 node-positive aTTom patients. They found a significant benefit from extended tamoxifen for patients classified as BCI (H/I)-High (HR 0.35; 95% CI 0.15–0.86): The risk of recurrence was 27.0% and 37.2% for patients treated with 10- and 5-year tamoxifen, respectively, demonstrating a significant absolute 10.2% reduction of the risk of recurrence. In contrast, there was no significant benefit from an additional 5 years of tamoxifen in patients classified as BCI (H/I)-Low (HR 1.07; 95% CI 0.69–1.65). As described by Simon et al., level 1B classification for the clinical utility of a biomarker requires reproducibility of the results in at least two independents prospective–retrospective studies. BCI (H/I) can thus be considered a biomarker of response to extended endocrine therapy. Despite these encouraging results, the routine use of such assays is limited by availability and prohibitive costs. They are not yet recommended for guiding therapy of ER + BC beyond 5 years from diagnosis [21, 22].

The results of our study suggest that also CTS5 can predict the response to extended endocrine therapy and should hence be taken into account in settings with difficult access to genomic signatures.

Limitations of this study are its pure retrospective design, the small number of patients prescribed the extended endocrine therapy, and the relatively short median follow-up that could have missed some LDR which are yet to develop. Moreover, the prescription of an extended endocrine regimen was based on the clinicians’ choice after the assessment of the risk factors taken singularly, since no integration tool was available in the period considered. Hence, to correctly assess the predictive ability of CTS5, we recommend testing the score on a wider cohort until a prospective analysis can be carried out. The point of strength is the inclusion of pre and postmenopausal patients, the majority of whom have been treated according to current guidelines, an aspect that enhances the applicability of the results of this study to everyday clinical practice.

## Conclusions

Retrospective risk stratification according to CTS5 appeared to be predictive of the response to extended endocrine therapy in our population of real-life pre and postmenopausal patients. Our results suggest its use in the clinical practice to better tailor the prescription of the extended adjuvant endocrine regimen.
